# Where the congenital heart disease meets the pulmonary arterial hypertension, *FLNA* matters: a case report and literature review

**DOI:** 10.1186/s12887-020-02393-2

**Published:** 2020-11-03

**Authors:** Xiaoxian Deng, Shanshan Li, Qiu Qiu, Bowen Jin, Menghuan Yan, Yuanpin Hu, Yang Wu, Hongmei Zhou, Gangcheng Zhang, Xuan Zheng

**Affiliations:** 1 Congenital Heart Disease center, Wuhan Asia Heart hospital, 753 Jinghan Ave, 430022 Wuhan, China; 2 Laboratory of Molecular Cardiology, Wuhan Asia Heart hospital, 753 Jinghan Avn, 430022 Wuhan, China; 3 Imaging center, Wuhan Asia Heart hospital, 753 Jinghan Ave, 430022 Wuhan, China

**Keywords:** Pulmonary arterial hypertension, Congenital heart disease, Filamin A

## Abstract

**Background:**

Pediatric patients with genetic disorders have a higher incidence of pulmonary arterial hypertension (PAH) regardless of their heart defects. Filamin A (*FLNA*) mutation is recently recognized to be associated with pediatric pulmonary disorders, however, the clinical courses of PAH related to the mutation were reported in limited cases. Here, we presented a case and pooled data for better understanding of the correlation between *FLNA* mutation and pediatric PAH.

**Case presentation:**

The patient was a 8-month-old female with repeated episodes of pneumonia. Physical examination revealed cleft lip, cleft palate and developmental retardation. Imaging examination showed a small atrial septal defect (ASD), central pulmonary artery enlargement, left upper lobe of lung atelectasis, and pulmonary infiltration. Genetic test showed she carried a de novo pathogenic variant of FLNA gene (c.5417-1G > A, p.-). Oral medications didn’t slow the progression of PAH in the patient, and she died two years later.

**Conclusions:**

*FLNA* mutation causes rare but progressive PAH in addition to a wide spectrum of congenital heart disease and other comorbidities in pediatric patients. We highly recommend genetic testing for pediatric patients when suspected with PAH. Given the high mortality in this group, lung transplantation may offer a better outcome.

## Background

Pediatric pulmonary arterial hypertension (PAH) is a rare disease with high mortality. Left-to-right shunting, lung diseases and genetic disorders are most common causes leading to PAH in children[1]. Filamin A (*FLNA*) is a 280-kD protein widely expressed in the body and regulating cell shape and migration. Among the broad range of diseases associated with *FLNA* mutation, lung diseases have been seen in most patients, such as pneumonia, and respiratory failure. In addition, PAH in pediatric patients with *FLNA* mutation was fatal despite of their congenital heart disease (CHD), and required early lung transplantation[2]. Here we report a female patient with *FLNA* mutation, who presented with recurrent pneumonia, arterial septal defect (ASD), mild developmental delay and rapidly progressive PAH.

## Case presentation

**Fig. 1:**
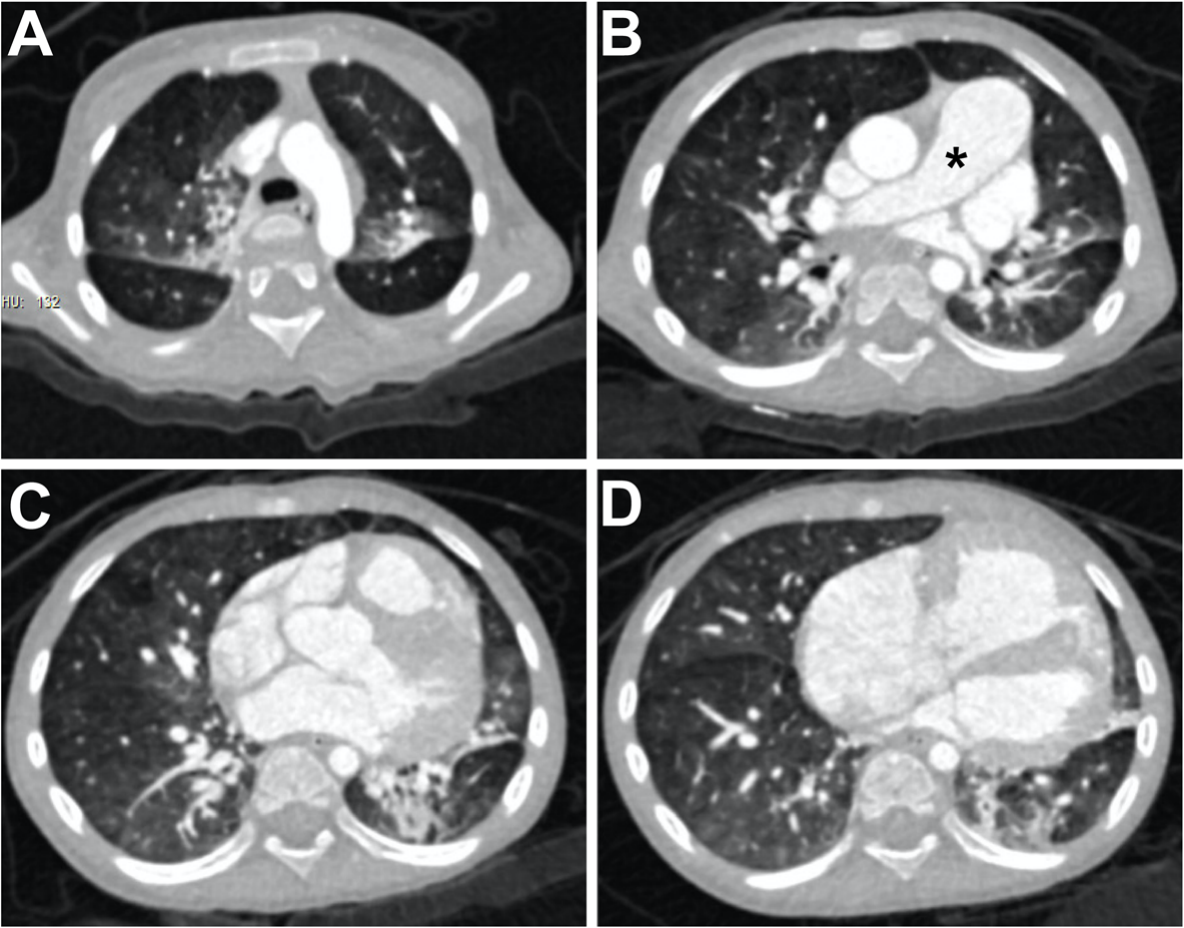
Chest CT. **a** Infiltration in both upper lobes of lung; **b** Main pulmonary artery was dilated (*). There was atelectasis in left upper lobe of lung; **c** Slightly infiltration in lower lobes; **d** Dilated right atrium.

An 8-month-old female patient was referred to our center due to severe cough, short of breath, fatigue and fever. The patient had nine episodes of pneumonia and cardiomegaly since she was two-month-old. Physical examination revealed cleft lip, which was surgical repaired when she was 6 months old, and cleft palate. Her finger oxygen saturation was 94%. Transthoracic echocardiography showed there was a 0.5 × 0.6 cm ASD with a 2.4 cm right atrium. Laboratory test showed NT-proBNP was 963 pg/ml. Some of autoimmune antibodies, including dsDNA-antibody, SSA/Ro 60kD antibody, anti-cardiolipid antibody, and anti-β2GPI antibody, were positive. Erythrocyte sedimentation rate (ESR) and C-reaction protein (CRP) were normal. IgG was slightly elevated at 18.40 g/L, and C3 was 0.83 g/L. Significantly increased pulmonary vascular resistance (PVR, 17 WU) was seen in her first right heart catheterization despite of the slightly increased pulmonary artery pressure (PAP, 38/17/24 mmHg). Oral furosemide and antisterone were given since then. She was also suggested to inhale oxygen at home even though she maintained her daily activities without additional requirement of oxygen. The patient was re-hospitalized several times because of recurrent pneumonia and heart failure thereafter. Her finger oxygen saturation dropped to 75% at lowest, and stayed at 95% or higher when given nasal catheter oxygen inhalation. Hemodynamic parameters turned worse in the second measurements, where PAP increased along with PVR (PAP, 100/50/67 mmHg; PVR, 42 WU). Further examination included chest computed tomography (CT) scan. CT showed infiltration in upper lobes at both sides (Fig. 1a, b), and lung atelectasis in left upper lobe (Fig. 1b). Pulmonary artery and right atrium were significantly dilated (Fig. 1b, star; d). No thrombosis was seen in pulmonary artery. The patient and her parents received whole exome sequencing test. A new splicing variant (exon34: c.5417-1G > A, p.-) in the *FLNA* gene was found only in the patient. Diuretics, dopamine, and oral Bosentan (12.5 mg twice daily) were used to relieve her symptoms. No intubation or other advanced life supports were required during hospitalizations. Patient’s family refused any further intervention during her last hospitalization at age of 2 years. She became significantly cyanosis after last discharge. Unfortunately, the patient didn’t response well to medication therapy, and she died from a severe pneumonia 5 months later.

## Discussion and conclusions

PAH is a clinical symptom characterized by increased pulmonary artery pressure more than 25 mmHg. Pediatric PAH shares similarities with adult PAH in some etiology. However, specialists have addressed that pediatric patients have higher prevalence of idiopathic PAH, PAH associated with congenital heart disease (CHD), and pulmonary disorders [3]. With the attempt to explore mechanism underlying, next generation sequencing reveals the genetic defects associated with pediatric PAH.

*FLNA* gene was firstly related to neurologic disorder defect periventricular heterotopia (PVNH) in 1998 [4]. A broad range of diseases were observed with *FLNA* mutation thereafter, such as otopalatodigital syndrome (OPD) [5], frontometaphyseal dysplasia (FMD) [6], and Melnick-Needles syndrome (MNS) [5], FG syndrome (FGS), chronic idiopathic intestinal pseudoobstruction (CIIP) [7], cardiac valvular disease (CVD) [8], and others. However, lung disease was noticed in patients with *FLNA* mutation first by de Wit MC, et al. in 2010 [9]. Patients with lung disease related to *FLNA* mutation had higher incidence of pneumonia, lung developmental defects and respiratory failure, however, PAH were uncommon [10–12]. Among the reported cases, there were 19 of them having early onset PAH (including this case). Their clinical characteristics are summarized in Table 1. Developmental delay was observed in 6 patients, while CHD were seen in all. Fourteen patients had surgical correction of CHD, 6 of which had lung transplantation at the same time. Only one patient died after lung transplantation, nonetheless, mortality among pediatric PAH patients with *FLNA* mutation is as high as 35%.

**Table 1:** Summary of pediatric PAH associated with *FLNA* mutation

	Mutation	Sex	Age at diagnose	CHD	Chest CT	Lung transplantation	Medicine	Outcome
Masurel-Paulet 2011[13]	mosaic nonsense mutation c.994delG(p.K331X)	male	3 months	PDA	Bilateral atelectasis; lung cysts; tracheobronchomalacia; pulmonary emphysema; congenital lobar emphysema;	N	None	ND
Reinstein 2013 [14]	De novo c.2193C > A (p.Tyr731X)	female	6 months	PDA	Areas of focal hyperinflation associated with minimal patchy atelectasis	N	Sildenifil	ND
	De novo deletion of exons 2,5 and 13	female	18 months	VSD	N/A	N	Bosentan	ND
	De novo c.5498_5504delCACCCACinsAC	male	2 months	ASD;VSD; PDA	N/A	N	None	Died
Lord 2014 [12]	Truncating filamin A mutation(c.5683G-T, p.G1895*)	female	4 months	ASD	Bilateral pulmonary atelectasis and cysts, tracheobronchomalacia, Areas of hyperinflation alternating with heterogeneous areas of atelectasis; alveolar simplification	N	Inhaled nitric oxide; sidenafil; bosentan	ND
Eltahir 2016 [13]	c.3153dupC in exon 21	female	2 months	PDA	Bilateral lung emphysema with basal atelectasis; bronchospasm	N	None	Died
Burrage 2017 [15]	Heterozygous c.4596dupG (p.Ser1533Glufs*12) (de novo)	female	4 months	PFO, PDA	Multifocal atelectasis; perinflation and hyperlucency; atelectasis; central pulmonary artery enlargement; tracheobronchomalacia	Y	Sildenafil	Alive
	Heterozygous c.5290G > A (p.Ala1764Thr) (de novo)	female	2 months	PFO, PDA, VSD	Perinflation hyperlucency; atelectasis; central pulmonary artery enlargement; tracheobronchomalacia	Y	Sildenafil	Died
	Heterozygous c.4446_4447dupAT(p.Leu1483Tyrfs* 19) (de novo)	female	1month	PFO,PDA	Perinflation hyperlucency; atelectasis; central pulmonary artery enlargement; tracheobronchomalacia	Y	Sildenafil	Alive
	Heterozygous c.4617_4618delGC(p.Leu1540Alafs*)	female	2 months	PFO, PDA	Perinflation hyperlucency; atelectasis; central pulmonary artery enlargement; tracheobronchomalacia	Y	Sildenafil	Alive
	Heterozygous c.6585dupT (p.Pro2196Serfs*3) (de novo)	female	7 montsh	PFO;PDA	Perinflation hyperlucency; atelectasis; central pulmonary artery enlargement; tracheobronchomalacia	Y	Sildenafil	Alive
	Heterozygous c.2807A > G (p.Lys936Arg) (VUS)	female	5 months	PFO, PDA	Perinflation hyperlucency; atelectasis; central pulmonary artery enlargement; tracheobronchomalacia	Y	Sildenafil	Alive
Shelmerdine 2017 [10]	Heterozygous for c.88delG, p.(Ala30fs)	female	ND	PDA;PFO	Left lung hyperinflation; interstitial thickening in left; mediastinal shift to the right; right lobe consolidation	N	None	Died
	Heterozygous for c.6496dupA, p. (lle2166fs)	female	ND	PDA	Progressive right lung hyperinflation; mediastinal shift to the left; right upper and middle lobe over inflation; coarse septal thickening; lower lobe atelectasis; patchy ground glass changes in lower lobes	N	Sildenafil	Alive
	Heterozygous for c.2190_2193delTTAC, p(tyr731fs)	female	ND	ASD;PDA	Right upper lobe hyperinflation; right middle lobe and left lower lobe atelectasis; right upper and middle, left upper lobe over-inflation; coarse septal thickening; lower lobe atelectasis	N	None	Alive
Kinane 2017 [16]	c.6577delC; p.Arg2193AlafsX14[R219AfsX14	female	7w	PFO; VSD; PDA	Wilson–Mikity syndrome (pulmonary dysmaturity syndrome)	N	None	ND
Sasaki 2018 [17]	Deletion c.6670-1delG	male	neonate	PDA	Bilateral dependent and subsegmental atelectasis, scattered opacity	N	None	Died
Cannaerts 2018 [18]	cis-located c.7921C > G,p.Pro2641Ala,c.7923delC,p.Tyr2642Thrfs*63	female	ND	ASD	ND	N	None	Died
This case	splicing c.5417-1G > A (exon 34)	female	22 months	ASD	Central pulmonary artery enlargement; left upper lobe atelectasis	N	Bosentan	Died

*FLNA* Filamin A; *CT* computed tomography; *PDA* patent ductus arteriosus; *VSD* ventricle septal defect; *ASD* atrial septal defect; *N* no; *Y* yes; *ND* Not provided

Interstitial lung disease (ILD) may cause PAH in pediatric patients, and *FLNA* mutation has been called for attention in ILD [17], but pediatric PAH patients with *FLNA* mutation don’t always present with characteristically pulmonary pathologic changes of ILD. Moreover, high prevalence of CHD in patients with *FLNA* mutation may confuse the real cause of the rapidly progressive PAH [19]. In our experience, genetic testing is more helpful to offer early-stage and accurate diagnose. Moreover, lung transplantation would bring higher survival in these patients based on previous reports.

## Data Availability

The datasets used in current study are available from the corresponding author on reasonable request.

## Abbreviations

FLNA: Filamin A
CHD: Congenital heart disease
ASD: Atrial septal defect
VSD: Ventricle septal defect
PDA: Patent ductus arteriosus
PFO: Patent foramen ovale
RHC: Right heart catheterization
PAH: Pulmonary arterial hypertension
NT-proBNP: N-terminal prohormone of brain natriuretic peptide
ESR: Erythrocyte sedimentation rate
CRP: C-reaction protein
PVR: Pulmonary vascular resistance
WU: Wood unites
PAP: Pulmonary artery pressure
CT: Computed tomography
PVNH: Periventricular heterotopia
OPD: Otopalatodigital syndrome
FMD: Frontometaphyseal dysplasia
MNS: Melnick-Needles syndrome
FGS: FG syndrome
CIIP: Chronic idiopathic intestinal pseudoobstruction
CVD: Cardiac valvular disease
